# A resource scheduling method for reliable and trusted distributed composite services in cloud environment based on deep reinforcement learning

**DOI:** 10.3389/fgene.2022.964784

**Published:** 2022-10-10

**Authors:** Lei Yu, Philip S. Yu, Yucong Duan, Hongyu Qiao

**Affiliations:** ^1^ Department of Computer Science, Inner Mongolia University, Hohhot, China; ^2^ Department of Computer Science, University of Illinois at Chicago (UIC), Chicago, IL, United States; ^3^ College of Computer Science and Technology, Hainan University, Haikou, China

**Keywords:** composite services, container cloud, deep reinforcement learning, service scheduling, artificial intelligence

## Abstract

With the vigorous development of Internet technology, applications are increasingly migrating to the cloud. Cloud, a distributed network environment, has been widely extended to many fields such as digital finance, supply chain management, and biomedicine. In order to meet the needs of the rapid development of the modern biomedical industry, the biological cloud platform is an inevitable choice for the integration and analysis of medical information. It improves the work efficiency of the biological information system and also realizes reliable and credible intelligent processing of biological resources. Cloud services in bioinformatics are mainly for the processing of biological data, such as the analysis and processing of genes, the testing and detection of human tissues and organs, and the storage and transportation of vaccines. Biomedical companies form a data chain on the cloud, and they provide services and transfer data to each other to create composite services. Therefore, our motivation is to improve process efficiency of biological cloud services. Users’ business requirements have become complicated and diversified, which puts forward higher requirements for service scheduling strategies in cloud computing platforms. In addition, deep reinforcement learning shows strong perception and continuous decision-making capabilities in automatic control problems, which provides a new idea and method for solving the service scheduling and resource allocation problems in the cloud computing field. Therefore, this paper designs a composite service scheduling model under the containers instance mode which hybrids reservation and on-demand. The containers in the cluster are divided into two instance modes: reservation and on-demand. A composite service is described as a three-level structure: a composite service consists of multiple services, and a service consists of multiple service instances, where the service instance is the minimum scheduling unit. In addition, an improved Deep Q-Network (DQN) algorithm is proposed and applied to the scheduling algorithm of composite services. The experimental results show that applying our improved DQN algorithm to the composite services scheduling problem in the container cloud environment can effectively reduce the completion time of the composite services. Meanwhile, the method improves Quality of Service (QoS) and resource utilization in the container cloud environment.

## 1 Introduction

With the rapid development and popularity of the Internet, the number of network users is also increasing, but the resources of the data center decreased relatively. The development of cloud computing technology has led to a great convenience of information processing, and users can obtain reliable services through the cloud platform on a large number of data centers. However, as the composite service requested by the users are complex and diversified, the number of requests is increasing. Especially in the field of bioinformatics, biomedical research relies on a large amount of genomic and clinical data. Biomedical companies form a data chain on the cloud, and they provide services and transfer data to each other to form composite services. In such a dynamic environment, resource management and performance optimization have become a significant challenge for cloud and application providers, who not only consider user Quality of Service (QoS) but also consider the load balancing of the data center, resource utilization and problems such as energy consumption (Almansour and Allah, 2019). Therefore, an efficient and reasonable service scheduling method becomes essential for the cloud computing platform.

In addition, many cloud platforms currently use virtual machines as the underlying virtualization technology. Additional operating systems carried by virtual machines will bring performance losses to the cloud platform, and the startup speed of virtual machines is slow, so it is difficult for them to make rapid scaling responses to service load (Barik et al., 2016). As the virtualization technology at the operating system level, the container technology has minimal additional resource overhead, shorter startup and destruction time, and the performance of disk IO and CPU of the container is even close to that of the host (Joy, 2015). Therefore, it is considered to be a better solution for application distribution and deployment on the cloud platform (Bernstein, 2014). Most of the research on service scheduling is based on virtual machines, while the research on composite service scheduling based on container cloud environment is in the exploratory stage. Because the container has the characteristics of fast startup, strong migration ability, low-performance cost, and high resource utilization (Joy, 2015), it is of great value and significance to take the container as the virtualized computing resource of the cloud platform to solve the service scheduling problem. We need a model and an algorithm that can be applied to the container cloud environment to reduce the completion time of the composite service, satisfy the user service quality as much as possible, and improve the resource utilization target of the cloud platform.

Therefore, we proposed a novel composite service scheduling model and algorithm according to container instance mode which mixed reservation and on-demand. In addition, the DQN (Deep Q-Network) algorithm is improved by combining the three algorithms Dueling-DQN (Wang et al., 2016), Double-DQN (DDQN) (Van Hasselt et al., 2016), and Prioritized Experience Replay (PER) (Schaul et al., 2016). DDQN improved the training algorithm by decoupling action selection and value function evaluation. Although it is not entirely decoupled, it effectively reduced over-estimation and made the algorithm more robust. PER introduced a new learning mechanism to solve the sampling problem of experience replay and innovatively took Temporal Difference (TD) deviation as an essential consideration to ensure that important experience can be replayed first, and the priority experience replay was applied to DQN and DDQN. The learning efficiency is greatly improved. Dueling DQN is an improvement of the neural network structure, which can decouple the value and advantages of the DQN. Although the value function and the advantage function can no longer be perfectly represented as the value function and the advantage function in semantics, the accuracy of the strategy evaluation was improved, and it can be combined with other algorithms due to the strong versatility. Thus, the management of the DQN algorithm is improved. From the three levels of training algorithm, learning mechanism, and neural network structure, three improvements have been made based on DQN, but its implementation is more complex than these three algorithms. The improved DQN algorithm is used as the scheduling decision method under our model to reduce the completion time of the composite service and improve the user QoS and resource utilization of the cloud platform.

The contributions of this paper include: A new composite service scheduling model is built for container instance mode which mixed reservation and on-demand. The model considers many features, such as container storage, computing speed, network bandwidths and data streams of service output, etc. Furthermore, the model is suitable for Map-Reduce based services in distributed environments.

A new composite service scheduling algorithm is proposed, which can effectively reduce the completion time of the composite services. Meanwhile, the method improves Quality of Service (QoS) and resource utilization in the container cloud environment.

## 2 Related work

Cloud computing technology has greatly promoted the transformation of various industries and the development of technological innovation. With the advancement of medical technology, the field of biomedicine has ushered in the era of big data (Yang et al., 2021). The application of cloud computing in biomedicine is becoming more and more perfect. Myers et al. (2020) developed an R package, LDlinkR, which leverages the computing resources of the cloud by harnessing the storage capacity and processing power of the LDlink web server to calculate computationally expensive LD statistics. Service scheduling, as an effective method to satisfy Quality of Service (QoS), which can rationally allocate resources and reduce energy consumption in cloud environment, has always been a research hotspot of scholars in various fields (Kyaw and Phyu, 2020). At the same time, scheduling in the cloud environment is a multi-constraint, multi-objective and multi-type optimization problem (Chen et al., 2019). Some traditional scheduling algorithms, such as Round-Robin (RR) scheduling algorithm and Least Connection (LC) algorithm, do not consider the actual load and connection status of the work node. Scheduling problem can be regarded as the problem of finding the optimal one or a group of computing resources in a limited set of computing resources under the condition of satisfying multiple constraint objectives. Heuristic algorithm is the most widely used method to solve such combinatorial optimization problems (Bernstein, 2014). The common ones are Ant Colony (AC) algorithm, Particle Swarm Optimization (PSO) algorithm, Genetic Algorithm (GA), etc. Therefore, many scholars are solving the problem of service scheduling in cloud platforms by optimizing and improving heuristic algorithms.

Panwar et al. (2019) combined Technique for Order Preference by Similarity to an Ideal Solution (TOPSIS) algorithm and PSO algorithm to divide task scheduling into two phases, which reduces the makespan of tasks and improves resource utilization of cloud platform. Chen et al. (2019) modeled the cloud workflow scheduling problem as a multi-objective optimization problem that takes both execution time and execution cost into account, and proposed a multi-objective ant colony system based on the co-evolutionary multi-population and multi-objective framework, in which two ant colony algorithms were adopted to deal with the two objectives, respectively. Cui and Xiaoqing (2018) proposed a workflow tasks scheduling algorithm based on a genetic algorithm. It plays an optimal role in the execution time of the optimal allocation scheme. George et al. (2016) adopted the Cuckoo Search algorithm to complete the assignment of tasks with the optimization goal of minimizing the computation time of tasks. Ghasemi et al. (2019) proposed a workflow scheduling method based on the Firefly Algorithm (FA), aiming at minimizing the processing time and transmission cost of workflow.

Compared with traditional scheduling algorithms, heuristic algorithms have a stronger ability for exploration and optimization. The above improvements of heuristic not only inherited the advantages of heuristic algorithms in solving combinatorial optimization problems but also solved some problems of heuristic algorithms themselves to some extent. However, these algorithms still have some problems, such as the weight coefficients of resources according to subjective experiences, slow convergence, and easily falling into local optimal solutions.

Considering the uncertainty of user requests, the dynamic nature of computing resources, the heterogeneity of cloud platforms, and many other factors, it has higher requirements for cloud platform service scheduling strategy. In recent years, with the development of artificial intelligence-related technologies, Deep Reinforcement Learning (DRL) has shown strong perception and continuous decision-making ability when dealing with automatic control problems (Orhean et al., 2018), and many scholars have begun to apply it to resource allocation and service scheduling strategies in cloud environments. Li and Hu (2019) described job scheduling as a packing problem, used DRL algorithm to calculate the fitness of jobs and machine nodes, and selected reasonable machines for jobs according to the fitness. Finally, through experiments, it proved the superiority of deep reinforcement learning as a scheduling algorithm. Cheng et al. (2018) designed a two-level scheduler combining resource allocation and task scheduling based on Deep Q-Learning, which greatly reduced the energy consumption of the cloud platform while maintaining a low task rejection rate. Wei et al. (2018) proposed an intelligent QoS aware job scheduling framework based on Deep Q-Learning algorithm, which can effectively reduce the average response time of jobs under varying loads and improve user satisfaction. Meng et al. (2019) designed an adaptive online scheduling algorithm by combining reinforcement learning with DNN, which significantly improved the scheduling efficiency of server-side task queues. Ran et al. (2019) used the Deep Determining Policy Gradient (DDPG) algorithm to find the optimal task assignment scheme meeting the requirements of the Service Level Agreement (SLA). Zhang et al. (2019) proposed a parallel execution multi-task scheduling algorithm based on deep reinforcement learning. And compared with least connection and particle swarm optimization, this algorithm significantly reduces the completion time of the job. Dong et al. (2020) proposed a task scheduling algorithm based on DRL, which can dynamically schedule tasks that have priority relationships in the cloud server, thus minimizing the task execution time and effectively solving the task scheduling problem in the cloud manufacturing environment.

Based on the above work, both the heuristic algorithm and deep reinforcement learning algorithm show their respective advantages in solving scheduling problems in cloud environments. However, there are still some problems that have not been considered in some references when solving scheduling problems in cloud platforms. References (Almezeini and Hafez, 2017; Li and Hu, 2019; Panwar et al., 2019) only discussed a single service type without discussing the diversity of services and the correlation between services. References (Cui and Xiaoqing, 2018; Xiaoqing et al., 2018) gave the corresponding weight coefficients of each resource through subjective experience. References (Zhang et al., 2019; Dong et al., 2020) did not take into account the transmission cost between resource nodes of the execution results of services in the actual scheduling process of composite services. In the actual environment, the data transmission time between sub-services affects the completion time and operation cost of composite services to some extent. With the increasing complexity of user requests and the increasing granularity of services, each service can be scheduled for parallel execution in multiple servers to reduce the response time of services and improve the quality of services for users. References (Orhean et al., 2018; Xiaoqing et al., 2018; Chen et al., 2019; Dong et al., 2021) did not consider the parallelism of services when discussing the problem of service scheduling. We compared some algorithms in Table 1.

**TABLE 1:** Summary of reviewed papers related to the task scheduling in the cloud computing.

Algorithm	Core issues to be solved	Algorithm idea	Advantage
Dueling-DQN Wang et al. (2016)	Solved the problem that in some states, action is of low importance to the overall result, and distinguished the change of Q value caused by action and state	Improved the architecture, the idea of advantage was added to evaluate the advantage function	Ensured that the relative ranking of the dominant functions of each action in this state remains unchanged
		Analyzed the advantages and disadvantages of state and action, respectively	Narrowed the range of Q value. Removed excess degrees of freedom. Improved the stability of the algorithm
DDQN Van Hasselt et al. (2016)	Solved the problem of overestimation in DQN algorithm	The idea of Double Q-learning is to reduce overestimations by decomposing the max operation in the target into action selection and action evaluation	More stable training results
			Reduced the error caused by variance
PER Schaul et al. (2016)	Changed the selection method of samples in experience replay	Improved the experience buffer training strategy	More robust
	Solved the problem of local optimization	More robust	Improved the performance of DDQN
	Offset the impact of sample distribution	Added weight to the original gradient update in SGD	Simple implementation
MOACS Chen et al. (2019)	Optimized execution time and cost	Two ant colonies are adopted to optimize execution time and execution cost, respectively	MOACS has better global search ability, particularly when dealing with large-scale workflows
		A new pheromone update rule is designed. The CHS is proposed to ensure the quality of the other objective	MOACS can generate a solution with similar WET but lower WEC than the other approaches
TOPSIS–PSO Panwar et al. (2019)	Improved the execution time, maximum completion time, resource utilization, processing cost, and transmission time in the process of task scheduling	The task scheduling is performed in two phases	Improved average resource utilization
		TOPSIS method calculates the RC of VMs with respect to each task	Low processing cost
		The PSO algorithm receives the calculated RC of each task which acts as FV of tasks (particles)	Reduced makespan for tasks
Workflow tasks scheduling optimization based on genetic algorithm Cui and Xiaoqing (2018)	Applicable to cloud computing environment combining task characteristics and resource characteristics	Assigned priority to each task	Reduced workflow scheduling cost
		Workflow tasks were divided into different levels, and a two-dimensional coding method was designed	
	Reduced the execution cost of workflow task scheduling	A new genetic crossover and mutation operation were designed to produce new different offspring, so as to increase population diversity	
FA Ghasemi et al. (2019)	Optimized the cost of executing the whole workflow and load balancing among workstations	The position of each firefly represents the feasible solution to a problem to be solved, and the brightness of the firefly represents the fitness of the firefly’s position	Minimized the processing time
		Each firefly flies towards a firefly that looks brighter than itself	Reduced transmission cost of workflow
An intelligent QoS-Aware Job Wei et al. (2018)	Met the QoS requirements of users	Learnt to make appropriate online job-to-VM decisions for continuous job requests directly from its experiences without any prior knowledge	Reduced the average response time of jobs under different loads. Improved user satisfaction
Scheduling and resource management algorithm for multi-user mobile-edge computing systems Meng et al. (2019)	The problem of delay-sensitive task scheduling and resource (e.g., CPU, memory) management on the server side in multi-user MEC scenario	Built a system that learns to manage resources directly from experience by using reinforcement learning with adaptive policy iteration represented *via* DNN.	Reduced average slowdown and average timeout period of tasks in the queue
		Designed a new reward function to reduce average slowdown and average timeout period of tasks in the queue	Improved the scheduling efficiency of server-side task queue
DDPG Ran et al. (2019)	Model free strategy for learning continuous action	DDPG combines the ideas of DPG and DQN	DDPG can run in a continuous action space
		It used the experience replay and delayed update target network in DQN	Solved the classical inverted pendulum control problem
		It can run in continuous action space based on DPG	Met service level agreements
MDTS Zhang et al. (2019)	The problem of scheduling jobs with scalable parallel tasks in general parallel computing systems, where there is a demand to determine the task placement plan with the goal of minimizing the job completion time, the load imbalance value, and the total cost	Within each task-specific branch, there is a fully connected layer and an output layer	Reduced the job completion time and optimized the load balancing problem. Improved task scheduling performance. MDTS is superior to the raw DRL algorithm
Data-dependent tasks re-scheduling energy efficient algorithm Xiaoqing et al. (2018)	Reduced energy consumption in the data center	Set the task priority to the sum of the upper and lower values of the task	Reduced energy consumption in the data center
		Used the task priority to calculate the critical path and critical resources of the task graph	
		Calculated the energy efficiency of each resource under the initial scheduling scheme	
DRL-based algorithms Islam et al. (2021)	Satisfied generalization to optimize multiple objectives while capturing or learning the underlying resource or workload characteristics	Two DRL-based agents (DQN and REINFORCE) DQN: An *ϵ*-greedy policy was used that selects the greedy action with probability 1 − *ϵ* and a random action with probability *ϵ*	Reduced both the total cluster VM usage cost and the average job duration
		REINFORCE: It worked by utilizing Monte Carlo roll-outs. After the collection step, the algorithm updates the underlying network using the updated policy gradient	
		Trained them as scheduling agents in the TF-agent framework	
Sharer Liang et al. (2020)	Improved the efficiency of resource management in CMfg	The proposed model transformed metrics generated from the individual needs of multiple users into a multiobjective reward	Adapt to different conditions
		Proposed a blacklist mechanism and a narrow baseline to improve the learning performance of RL	Converged quickly

In addition, most of the above studies took virtual machines as virtualized computing resources to study the problem of service scheduling, while containers have the advantages of simple deployment and fast startup speed, so it is of certain research significance and value to discuss the problem of service scheduling based on the container cloud environment.

## 3 System model

### 3.1 Problem description

Based on the container cloud environment, this section focuses on the scheduling method of composite services. In the initialization stage, a certain number of host nodes are set, and each host node initializes: 1) a certain number of reserved container instances with different configurations; 2) a certain number of on-demand containers. In the reserved mode, the container instance is in the startup state and uses the allocated resources for the scheduled services at any time. The container in on-demand mode is dormant initially and takes a period of time to be started. The composite service is defined as the three-level structure of “composite service, sub-service, instance.” As the basic scheduling unit, the sub-service instance is scheduled to be executed in the container, which in essence represents the number of parallel execution of sub-services. In addition, the scheduling of sub-service instances and the starting of containers in on-demand mode are determined by the service scheduling algorithm.

### 3.2 Problem constraints

A composite service consists of multiple sub-services (hereinafter referred to as “Services”) that have an association relationship, including the order of prior execution and data dependencies among the services. In addition, each service includes one or more service instances, and each service instance of the same service has the same physical performance requirements. A composite service can be represented by a directed acyclic graph, i.e., *CS* = (*SVC*, *E*), where the finite set *SVC* = {*svc*_1_, …, *svc*_*m*_} indicates that a composite service contains *m*(*m* ≥ 1, *m* ∈ *N* ^+^) services. Each service has *n*(*n* ≥ 1, *n* ∈ *N* ^+^) service instances, denoted as ${svc}_{i}=\left\{{st}_{i}^{1},\ldots ,{st}_{i}^{n}\right\}\left(i\in m\right)$ . The set of directed edges *E* = {(*svc*_*i*_, *svc*_*j*_)∣1 ≤ *i*, *j* ≤ *m*, *i*, *j* ∈ *N* ^+^} describes the relationship between services, (*svc*_*i*_, *svc*_*j*_) means that *svc*_*i*_ is the predecessor service of *svc*_*j*_, and *svc*_*j*_ is called the successor service of *svc*_*i*_. Only after all service instances of all precursor services of *svc*_*j*_ have been executed, *svc*_*j*_ is allowed to be scheduled and executed. The service without the precursor service is called the start service *svc*_*start*_, and each composite services has at least one start service. Service without successor services is called end service *svc*_*end*_. Similarly, each composite service has at least one end service. Each Roman character (e.g., I, II) represents the number of service instances contained in the corresponding service. This scenario is prevalent for Map-Reduce algorithms in distributed environments.

Each service instance will be scheduled to a container, and each service contains multiple service instances, which means that each service can be executed by multiple containers together. The characteristic definition of service *svc*_*i*_ can be denoted by Eq. 1, where *cpu*_*i*_, *mem*_*i*_, *disk*_*i*_ represent the physical performance requirements of service *svc*_*i*_, such as CPU, memory, and disk storage, respectively. *length*_*i*_ denotes the length of the result data after the completion of the service execution; ${inst_{n}um}_{i}$ denotes the number of service instances of service *svc*_*i*_; *duration*_*i*_ represents the expected execution time of the subservice *svc*_*i*_.

$${svc}_{i}=\left\{{cpu}_{i},{mem}_{i},{disk}_{i},{length}_{i},{inst\text{\_}num}_{i},{duration}_{i}\right\}\left(i\in n\right)$$ (1)

As the smallest scheduling unit in a composite service, the service instances have the same physical resource requirements as the service it belongs to. All instances of the same service can be executed in parallel, and instances of each service are able to execute different binary files for Map-Reduce scenarios. Eq. 2 defines the *kth* service instance of *svc*_*i*_.

$${st}_{i}^{k}=\left\{k,{cpu}_{i},{mem}_{i},{disk}_{i},{length}_{i},{duration}_{i}\right\}\left(i\in n,k\in m\right)$$ (2)

### 3.3 Resource model

In the cloud platform, physical hosts are the infrastructure that truly provides physical resources such as CPU and memory for containers and services. All hosts in a host cluster are denoted as *H* = *h*_1_, …, *h*_*P*_, where *p* is the number of hosts in the cluster. *h*_*x*_(*x* ∈ *p*) represents the *xth* host in the host cluster, and the definition of *h*_*x*_ is shown in Eq. 3.

$$\begin{array}{cc} h_{x}= & \left\{hid,{cpu\text{\_}cap}_{x},{mem\text{\_}cap}_{x},{disk\text{\_}cap}_{x},{bw\text{\_}cap}_{x},\right.\\ & \left.{container\text{\_}num}_{x},{cpu}_{x},{mem}_{x},{disk}_{x},{bw}_{x}\right\}\left(x\in p\right) \end{array}$$ (3)

where *hid* represents the unique ID of the host. And *cpu*_*cap*_*x*_, *mem*_*cap*_*x*_, *disk*_*cap*_*x*_, *bw*_*cap*_*x*_, respectively represent the CPU capacity, memory capacity, disk storage capacity, and bandwidth capacity of the host. *container*_*num*_*x*_ represents the maximum number of containers that can be allocated by the host *h*_*x*_. *cpu*_*x*_, *mem*_*x*_, *disk*_*x*_, *bw*_*x*_ respectively represent the remaining amount of the host’s CPU, memory, disk storage, and bandwidth.

In addition, all containers in the cluster can be represented by the set *C* = {*c*_1_, …, *c*_*q*_}, where *q* is the number of containers. *c*_*y*_(*y* ∈ *q*) represents the physical performance state of the *yth* container, and the definition of *c*_*y*_ is shown in Eq. 4.

$$\begin{array}{cc} c_{y}= & \left\{{cid}_{y},{hid}_{y},{cpu\text{\_}cap}_{y},{mem\text{\_}cap}_{y},{disk\text{\_}cap}_{y}\right.,\\ & \left.{bw}_{y},{cpu}_{y},{mem}_{y},{disk}_{y},{act}_{y},{act\text{\_}time}_{y}\right\}\left(y\in q\right) \end{array}$$ (4)

where *cid*_*y*_ represents the container ID, which is the unique identifier of the container. *hid*_*y*_ represents the host ID to which the container *c*_*y*_ belongs. *cpu*_*cap*_*y*_, *mem*_*cap*_*y*_, *disk*_*cap*_*y*_, *bw*_*y*_ respectively represent the CPU capacity, memory capacity, disk capacity, and bandwidth capacity of the container *c*_*y*_. *cpu*_*y*_, *mem*_*y*_, *disk*_*y*_, respectively represent the remaining amount of the container’s CPU, memory, and disk during operation. *act*_*y*_ is the judgment flag, which indicates whether the container *c*_*y*_ is already in the state of the host. If *act*_*y*_ = 1, means that the container *c*_*y*_ is in the running state, and *act*_*y*_ = 0 means that the container *c*_*y*_ is in the dormant state. *act*_*time*_*y*_ represents the startup time of the container.

In order to compare and analyze resource utilization from three dimensions of CPU, memory, and disk, ${UST}_{i}^{k}$ is defined as the resource utilization after each service instance is scheduled. The definition of average resource utilization AVUST is shown in Eq. 5.

$$AVUST=\frac{\sum_{i=1}^{m}\sum_{k=1}^{n}{UST}_{i}^{k}}{number\;of\;service\;instances\;}$$ (5)

### 3.4 Scheduling model

Before all composite services are scheduled, the hosts and containers in the data center need to be initialized. In the initialization phase, a series of physical hosts with different configurations are first created, and each host is allocated with *container*_*num*_*x*_ containers, including different configurations of reserved and on-demand containers. The containers in the reservation mode can run the scheduled service instances at any time based on the allocated resources. The containers in the on-demand mode are in the dormant state by default, which occupies a certain amount of physical resources, but there are no remaining amount of resources. The resource state of the containers in the on-demand mode is shown in Eq. 6.

$$\left\{\begin{array}{c} {cpu\text{\_}cap}_{y}>0\;\\ {mem\text{\_}cap}_{y}>0\;\\ {disk\text{\_}cap}_{y}>0\;\\ {bw}_{y}>0\;\\ {cpu}_{y}=0\;\\ {mem}_{y}=0\;\\ {disk}_{y}=0\; \end{array}\right.$$ (6)

Constraints must be satisfied to schedule the service to the container for execution. When the service instance ${st}_{i}^{k}$ is scheduled to the container *c*_*y*_, the physical resource requirements of the service instance ${st}_{i}^{k}$ must not be greater than the corresponding physical resource capacity of the container *c*_*y*_, otherwise it will wait for the right resources to execute. Therefore, the constraint condition that needs to be met to dispatch the service instance ${st}_{i}^{k}$ to the container *c*_*y*_ is shown in Eq. 7.

$$\left\{\begin{array}{c} {cpu}_{k}\leq {cpu\text{\_}cap}_{y}\;\\ {mem}_{k}\leq {mem\text{\_}cap}_{y}\;\\ {disk}_{k}\leq {disk\text{\_}cap}_{y}\; \end{array}\right.$$ (7)

When a service *svc*_*i*_ is ready, all service instances of the service can be scheduled to the containers for execution one by one within the same scheduling time window. However, the resource status of the container changes from time to time as the service scheduling progresses. When the service instance is scheduled to the appropriate container, it will not be executed immediately. Because the following three steps are required:

(1) First, the status of the selected container needs to be determined. If the container has already been started, that is, *act*_*y*_ = 1, then ignore this step. Otherwise, *act*_*y*_ = 0, start the container, which will consume the time of *act*_*time*_*y*_.
(2) After the completion of step one, it is necessary to wait for the execution result of the precursor service to be transmitted to the container. The data transmission time is related to the result data length after the execution of the precursor subservice, the bandwidth of the container, and the host. Since the precursor service has multiple service instances, each service instance will be scheduled to run in a container. It can be understood that each service can be scheduled to run in multiple containers, so it is necessary to calculate the minimum transmission time of the result data from the container scheduled by the precursor service to the container where the current service instance is located. The data transmission time between containers in the same host is negligible. The data transmission time between different hosts is directly related to factors such as container bandwidth and data length. The data transmission time is shown in Eq. 8.

$$\begin{array}{cc} transTi^{k}\left(c_{u},c_{v}\right) & =\left\{\begin{array}{c} 0,\;\;\;\;\;\;\;u=v\;or\;{hid}_{u}={hid}_{v}\;\\ ratio,\;\;other\; \end{array}\right.\\ ratio & =\frac{{length}_{i}}{\;\min\;\left({bandwidth}_{u},\;{bandwidth}_{v}\right)} \end{array}$$ (8)

(3) In addition to the data transmission time, it is necessary to wait for the remaining amount of the physical resources of the container to meet the physical resource requirements of the service instance itself. Record the waiting resource time of the service instance ${st}_{i}^{k}$ in the container *c*_*y*_ as $wr_{i}^{k}$ .

Based on the above three steps, it can be concluded that after the service instance ${st}_{i}^{k}$ is scheduled, the period before execution is the total waiting time of the service instance ${TW}_{i}^{k}$ :

$${TW}_{i}^{k}=\left\{\begin{array}{cc} transTi^{k}+wr_{i}^{k},\; & \;\;\;{act}_{y}=1\\ {act\text{\_}time}_{y}+transTi^{k}+wr_{i}^{k},\; & \;\;\;{act}_{y}=0 \end{array}\right.$$ (9)

As mentioned above, the execution of the service is finished when all the instances of the service *svc*_*i*_ are executed. Therefore, the response time *T*_*i*_ of the service *svc*_*i*_ should be denoted as:

$$T_{i}=\underset{k}{\max}\left(T_{i}^{k}\right)$$ (10)

Taking the submission time of the composite services as the earliest start execution time *T*_*start*_ and the completion time *T*_*end*_ of the last service instance in the sub-service as the completion time of the composite service, thus the actual completion time *TC* of the entire composite service is denoted by Eq. 11.

$$TC=T_{\mathrm{end}}-T_{\mathrm{start}}$$ (11)

In order to denote the expected completion time of the composite services more conveniently, the composite service is divided into layers according to the execution order of the service. The start sub-service is placed in the first layer, and the end sub-service is placed in the last layer.

The service completion time of each level is the response time of the service with the longest response time in the level, as shown in Eq. 12, where *l* represents the level and *u* represents the number of services contained in the level.

$${TL}_{l}=\underset{u}{\max}\left(T_{i}\right)$$ (12)

Define the maximum expected completion time for an entire composite service as:

$$TE=2\underset{v}{\sum }{TL}_{l}$$ (13)

The interaction between the user and the cloud platform takes the whole composite service as the unit, and the user can set the desired QoS demand when sending the request. The completion time of the composite service is an important QoS indicator for users, so this paper takes the maximum expected completion time of the composite services *TE* as the user’s QoS demand. Eq. 14 indicates whether the user’s demand QoS can be met:

$$success\left(CS\right)=\left\{\begin{array}{cc} 1,\; & TC\leq TE\\ 0,\; & else \end{array}\right.$$ (14)

For cloud and service providers, the goal of service scheduling is to meet users’ QoS requirements as far as possible while completing service execution under the constraints of limited IaaS or PaaS resources, which needs to be implemented through an efficient online service scheduling algorithm.

## 4 Algorithm design and implementaion

### 4.1 Prioritized 3-deep Q-network

In the process of using DQN (Deep Q-Network), there will be a problem of overestimate (Liang et al., 2020). Therefore, in recent years, many scholars have proposed improved algorithms for DQN, including DDQN, Dueling DQN, distributed DQN, PER, etc. This section combines DDQN, Dueling DQN, and Prioritized Experience Replay three algorithms to improve DQN at the same time to construct Prioritized Dueling-DDQN (hereinafter referred to as Prioritized 3-DQN) algorithm. This algorithm avoids overestimation of DQN to a certain extent. At the same time, when updating the parameters of neural network, PER algorithm is used to replace the random sampling method in DQN and select the most effective learning samples from the sample memory to achieve the purpose of efficient learning.

The Prioritized 3-DQN algorithm also uses two neural networks with the same structure: the Eval network and the Target network. The Eval network is used to calculate the estimated *Q* value and can be updated in real time. The Target network is used to calculate the target *Q* value, and it is a temporarily frozen network. This article has made three improvements to DQN: two decoupling actions and one sampling method improvement. The specific descriptions are as follows:

(1) The output layer of the neural network is decoupled into two output streams, which output the current state value V and the action advantage function *A*, respectively, and then combine the state value V and the advantage function *A* to form the *Q* value. The advantage function refers to the degree of merit of the value that can be obtained by taking an action relative to the average value of the state for a particular state. In order to calculate the advantage function value corresponding to each action more conveniently, the average value of the advantage function value of all actions is set to 0. If the advantage function value corresponding to a certain action is greater than the average value in the state, then the advantage function value corresponding to the action is positive, and vice versa. At this time, the calculation method of the *Q* value is shown in Eq. 15, where *θ* represents the neural network parameter, *α* and *β* represent the output flow neural network parameters corresponding to the state value and the action advantage function, and *n* is the action dimension.

$$Q\left(s,a;\theta \right)=V\left(s;\alpha \right)+A\left(s,\;a;\beta \right)-\;\frac{\sum_{a^{\prime }}^{n}A\left(s,\;a^{\prime };\beta \right)}{n}$$ (15)

(2) Based on DQN, the overestimation problem is solved by decoupling the selection of target action and calculating the target *Q* value. When calculating the actual value of *Q*, the Eval network provides the action in the next environment state, and the Target network provides the *Q* value of this action.

The *Q* value. At this time, the update process of the neural network is shown in Eq. 16, where *θ* and *θ* ^−^ represent the Eval network and the Target network, respectively.

$$Q\left(s_{t},a_{t};\theta \right)\leftarrow Q\left(s_{t},a_{t};\theta \right)+\propto \left[r_{t}+\gamma Q\left(s^{\prime },a^{\max}\left(s^{\prime };\theta \right);\theta^{-}\right)-Q\left(s_{t},a_{t};\theta \right)\right]$$ (16)

(3) In the offline training phase of traditional DQN, the training samples are randomly selected from the experience replay pool without considering the priority relationship of the samples. However, different samples have different values, and the samples directly affect the training effect of the neural network. In order to improve the training effect of the neural network, it is necessary to determine a priority for each sample and conduct sampling according to the priority of the sample. As mentioned above, the Target network does not have the function of real-time updates. Therefore, as the Eval network is continuously updated, there will be a certain gap between the two networks while calculating the *Q* value. This gap is named the timing difference TD_Error . TD_Error can be represented by Eq. 17. The larger the TD_Error , the larger the gap between the current *Q* function and the target *Q* function, the more the neural network needs to be updated at this time, so TD_Error can be used to measure the value of the sample. In order to prevent the network from overfitting, samples can be drawn by probability. At this time, the probability of samples being drawn is shown in Eq. 18, where *ϵ* is a small value close to 0, which guarantees Samples with TD_Error of 0 may also have a chance to be drawn.

$$TD\text{\_}Error=r_{t}+\gamma Q\left(s^{\prime },\;a^{\max}\left(s^{\prime };\theta \right);\;\theta^{-}\right)-Q\left(s_{t},\;a_{t};\;\theta \right)$$ (17)

$$P\left(i\right)=\frac{p_{i}}{\sum p_{i}}$$ (18)

where, $p_{i}=\left|TD_{E}rror+\epsilon \right|$ . The process of our Prioritized 3-DQN algorithm is as follows:

Algorithm 1 Prioritized 3-DQN.

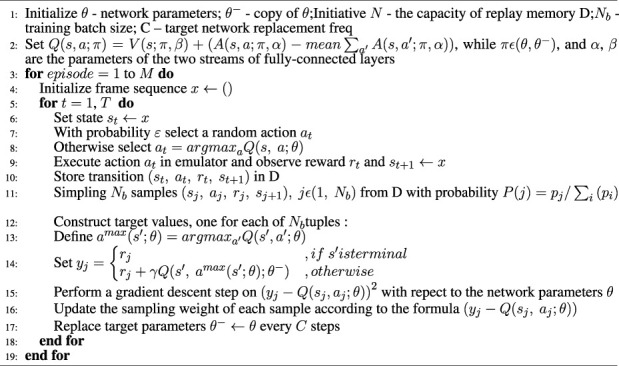

### 4.2 State space

When the service *svc*_*i*_is ready, the method selects an instance of *svc*_*i*_ each time ${st}_{i}^{k}$ and schedules it to a certain container. The environment status at this time is mainly determined by the physical relevant factors of the service instance ${st}_{i}^{k}$ , such as resource requirements, running status of the container cluster are determined. Therefore, the state space can be denoted by Eq. 19:

$$S_{i}^{k}=\left[{st}_{i}^{k},c_{1},{obs}_{c_{1}},{pre}_{c_{1}}^{{svc}_{i}},\ldots ,c_{q},{obs}_{c_{q}},{pre}_{c_{q}}^{{svc}_{i}}\right]$$ (19)

where ${obs}_{c_{y}}=\left[{que\text{\_}len}_{y},{cpu\text{\_}len}_{y},{mem\text{\_}len}_{y},{disk\text{\_}len}_{y}\right],\left(y\in q\right)$

Each value in the state space affects the scheduling decision of DRL, where ${st}_{i}^{k}$ represents the current service instance to be scheduled, which is represented by the aforementioned Eq. 2, and cy represents the resource state of the *yth* container in the cluster, as shown in Eq. 4. It should be noted that there is a one-to-many relationship between service instances and containers. Each service instance can only be completed by one container, but each container can be assigned multiple service instances. When the remaining physical resources of the container are insufficient and the resource requirements of the service instance are required, the newly scheduled service instance needs to be added to the services queue to be executed in the container. ${obs}_{c_{y}}$ is the running status of container *c*_*y*_, where *que*_*len*_*y*_ represents the length of the service instance queue to be executed in container *c*_*y*_, and *cpu*_*len*_*y*_, *mem*_*len*_*y*_, and *disk*_*len*_*y*_ respectively represent the sum of the CPU, memory, and disk storage space requirements of the waiting queue. The characteristic value ${pre}_{c_{1}}^{{svc}_{i}}$ represents the proportion of the result data length of the predecessor service of the current service instance in the container *c*_*y*_ after execution. For example *svc*_3_ has two predecessor services *svc*_1_ and *svc*_2_. Assume that the length of the result data after the execution of these two precursor services is 4 and 6, so only the service instance of *svc*_1_ is scheduled to the container *c*_1_. The service instance of *svc*_3_ is ${st}_{3}^{1}$ . When being scheduled, ${pre}_{c_{1}}=4/\left(4+6\right)=0.4$ .

### 4.3 Action space

During scheduling decision-making, a suitable container is selected for the service instance as the action in DRL, and the action space is all the containers that can be selected. Suppose that the data center contains *p* hosts {*h*_1_, …, *h*_*p*_} at a certain time, host *h*_*x*_ can assign at most *container*_*num*_*x*_ containers with different configurations. When service instance ${st}_{i}^{k}$ is ready to be scheduled, the agent in DRL can schedule it to any container in the cluster for execution, including all containers in reserved and on-demand modes. The action space at this time is shown in Eq. 20.

$$a_{\mathrm{num}}=h_{x}\times {container\text{\_}num}_{x}\;\left(x\in p\right)$$ (20)

### 4.4 Reward function

In order to enable the agent in DRL to learn effectively and obtain an effective scheduling strategy that optimizes the goal, a reasonable reward function needs to be designed to guide the learning process of the agent. In our model, in order to minimize the completion time and improve the user QoS and resource utilization of the cloud platform, this paper uses the difference between the expected execution time of the service instance and the waiting time. It then uses the ratio of the expected execution time as the reward for each scheduling. The value is as follows:

$$r_{i}^{k}=\frac{\left({durtation}_{i}-{TW}_{i}^{k}\right)}{{duration}_{i}}=1-\frac{{TW}_{i}^{k}}{{duration}_{i}}$$ (21)

Based on Eq. 21, the interval of reward value can be deduced as[ − *∞*, 1]. When the overall waiting time of the service instance ${TW}_{i}^{k}$ is 0, the scheduling reward reaches the highest value of 1; when the overall waiting time ${TW}_{i}^{k}$ is equal to the expected execution time, the reward value is 0; when the overall waiting time ${TW}_{i}^{k}$ is greater than the expected execution time, the reward value begins to show a negative value. The longer the waiting time for execution, the smaller the reward value, and the greater the punishment. Through the reasonable design of the reward function, DRL can learn an effective service scheduling policy.

## 5 Experimental results

### 5.1 Simulation experiment setup

This paper uses Alibaba Cluster Data V2018 (Alibaba, 2018) as the data set for the simulation experiment. The data set contains six files in CSV format, describing the status information of the physical machine cluster, container cluster, and batch processing tasks. The original data set has a huge amount of data. There is inevitably a problem of missing data, and the data set is scattered and difficult to operate. Therefore, it is necessary to preprocess the original data set to obtain more targeted and valuable data. During the experiment, the preprocessed batch job data needs to be parsed and mapped into a composite service entity. The comparison between the fields of the preprocessed batch_task table and the attributes of the service class is shown in Table 2.

**TABLE 2:** Table of data relation comparison.

Fields of batch_task table	Attributes of class service	Description
task_name	service_name	Service name
inst_num	inst_num	The number of instances
job_name	cs_name	The name of composite service
Duration	Duration	Expected execution time
plan_cpu	cpu	CPU cores requirements
plan_mem	mem	Memory requirements
Disk	Disk	Disk storage requirements
Length	Length	The length of result

This paper divides the experimental data set into two parts: the training data set and the test data set, as shown in Table 3. In this experiment, 5,832 pieces of data are selected as services from the batch_task table, forming a total of 1,036 composite services, including 38,586 service instances. At the same time, to fully verify the effectiveness of Prioritized 3-DQN as a scheduling algorithm, this paper sets up three test sets with different data volumes.

**TABLE 3:** Table of dataset settings.

Dataset name	The number of composite services	The number of services	The number of service instances
Training data set	1,036	5,832	38,586
Test data set1	345	1,500	12,320
Test data set2	426	2,200	18,020
Test data set3	512	2,780	25,200

In the initial stage of the simulation experiment, four hosts with different configurations are set, and each host contains container instances with different configurations and states. The relevant configuration of each container is shown in Table 4.

**TABLE 4:** Resource node settings.

Hosts	Containers	Detailed description
		(CPU cores; Memory capacity; Disk capacity; Bandwidth; Status)
Host 0	Container 0	4; 1.56; 10; 5; Running
	Container 1	4; 1.56; 10; 5; Stopped
	Container 2	8; 3.13; 18; 8; Running
Host 1	Container 3	4; 1.56; 10; 5; Stopped
	Container 4	8; 3.13; 18; 3; Running
	Container 5	8; 3.13; 18; 3; Stopped
Host 2	Container 6	4; 2.34; 12; 5; Stopped
	Container 7	8; 3.13; 18; 3; Running
Host 3	Container 8	4; 2.34; 12; 3; Running
	Container 9	8; 3.13; 18; 5; Stopped

In implementing the Prioritized 3-DQN algorithm, the parameter settings are shown in Table 5. Both the Eval network and the Target network contain three fully connected neural network hidden layers, the last layer of which is divided into two output channels: state value and action advantage function. The greedy coefficient *ɛ* is 0.9. Each time the neural network parameters are updated, it will increase by 0.0001. That is, when selecting the container for the service instance, the container with the largest *Q* value will be selected with a probability of 0.9, and the container will be randomly explored with a probability of 0.1. After 1,000 updates, the value of *ɛ* becomes 1, and random exploration is no longer performed when selecting a container, but only the container corresponding to the largest *Q* value is selected. *ϵ* is set to 0.001, which ensures that samples whose timing difference TD_Error is 0 will also have a chance to be sampled. The target network update frequency *C* is set to 30, which means that every 30 times the Eval network is updated, its network parameters are copied to the Target network.

**TABLE 5:** Algorithm parameter setting.

Parameter name	Value
The number of hidden layers	3
Activation function	ReLU
Greed index *ɛ*	0.9
Experience replay pool size *N*	3,000
Number of sample sets *N*_*b*	200
Learning rate *α*	0.001
Discount factor *γ*	0.9
*ϵ*	0.001
Target network update frequency *C*	30

### 5.2 Prioritized 3-deep Q-network training effect

**FIGURE 1:**
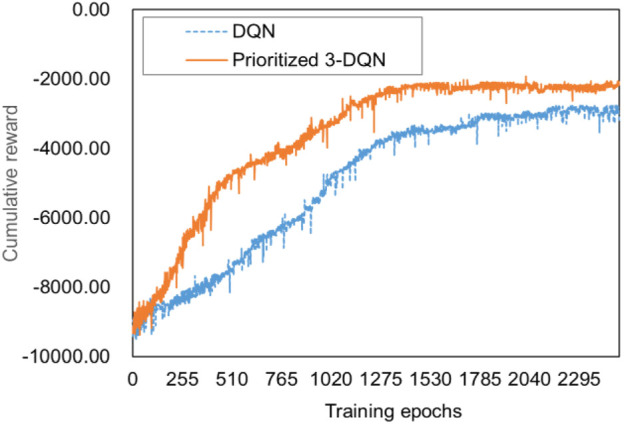
Training effect comparison chart.

The essence of deep reinforcement learning algorithm learning is to maximize the cumulative reward of the round as the optimization goal, so the training effect can be reflected by the trend of the cumulative reward as the value changes with the number of training rounds. In addition, the Prioritized 3-DQN scheduling algorithm proposed in this paper is improved based on the DQN algorithm. In order to evaluate the convergence and stability of the improved Prioritized 3-DQN scheduling algorithm, it is compared with the original DQN algorithm. After 2,500 rounds of training using the training data set, they finally reached their optimal training effects. Figure 1 is a comparison chart of training effects.

It can be seen from Figure 1 that as the number of training rounds increases, the cumulative reward values calculated by the two algorithms show a gradual upward trend. After a certain number of rounds, they have reached a stable trend, indicating Prioritized 3-DQN and DQN are reasonable as the scheduling algorithm of the composite service model proposed in this paper. However, from the perspective of convergence, our algorithm can obtain a higher cumulative reward value under the same number of training rounds. In addition, When the number of training epochs reaches around 1,600, the Prioritized 3-DQN scheduling algorithm starts to converge. The DQN starts to converge when the number of training rounds reaches about 2,200. Thus, the convergence speed of our algorithm is faster, and a higher cumulative reward value is obtained after the iteration is completed. This is because each time the weight parameters of the neural network are updated in our algorithm, the experience samples with larger time-series differences are selected first, so as to ensure the learning effect of the neural network. From the perspective of stability, Prioritized 3-DQN decouples the selection of the target *Q* value action and the target *Q* value calculation, thereby avoiding the problem of overestimation. Therefore, compared with the DQN rising trend, the upward trend of our results is slightly smoother and more stable. In general, our Prioritized 3-DQN is very suitable for composite service scheduling strategies. Compared with DQN, it has higher learning efficiency and can converge earlier to achieve better results.

### 5.3 Makespan comparison

**FIGURE 2:**
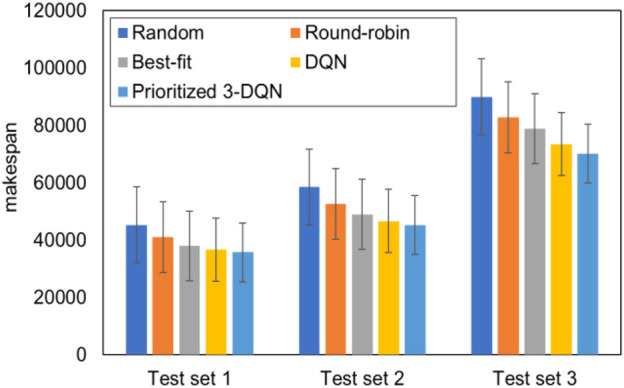
Comparison chart of average completion time of each test set with standard error.

To verify the generalization ability of Prioritized 3-DQN as a composite service scheduling algorithm, DQN and the four common scheduling algorithms mentioned above are respectively applied to the composite service model. In the process of the comparative experiment, three test sets were used for 20 experiments, the completion time of the composite service was calculated, and the average results were obtained. Figure 2 summarizes the average completion time obtained after 20 experiments on each of the three test data sets.

It can be seen from Figure 2 that the completion time of Prioritized 3-DQN on different test data sets is shorter than the results of the other four scheduling algorithms. Among them, the difference in completion time between DQN and Prioritized 3-DQN is smaller than the other three scheduling algorithms. The completion time of Prioritized 3-DQN on three data sets is about 3.32% less than that of DQN on average. The number of service instances in the three test sets increases sequentially. With the increase in the number of service instances, the increase in the completion time of the composite service under different scheduling algorithms is different, and the gap in completion time between Prioritized 3-DQN and the other four scheduling algorithms is more prominent. This means that the algorithm and DQN algorithm proposed in this paper are more adaptable than other algorithms in terms of completion time.

### 5.4 Quality of service comparison

**FIGURE 3:**
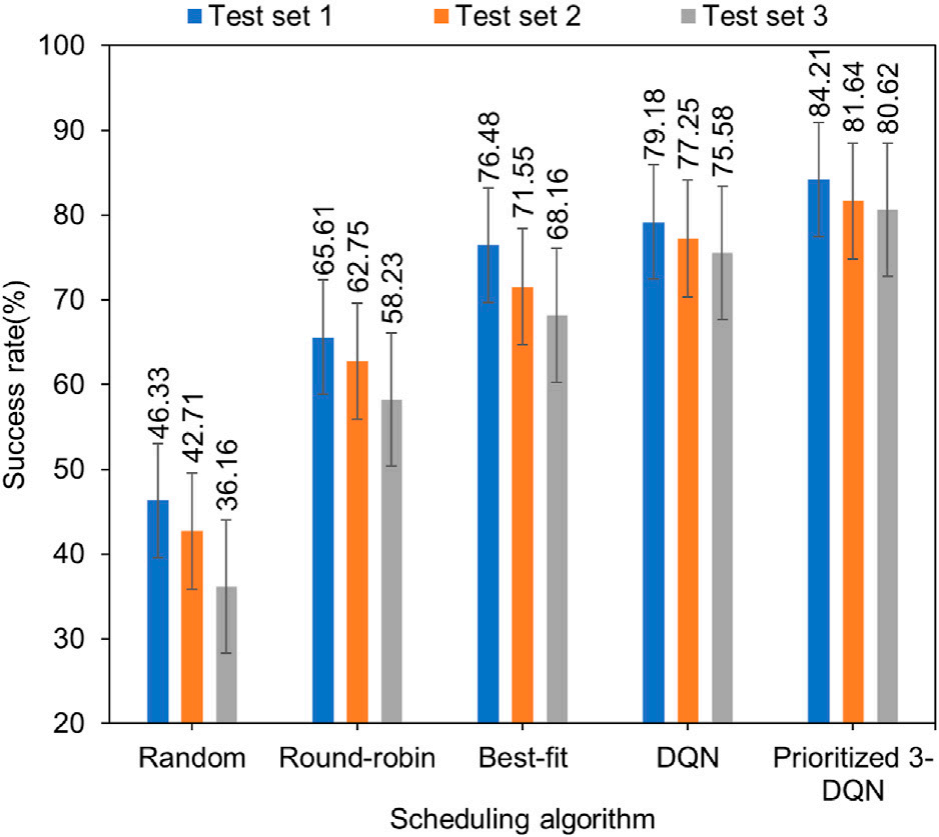
Comparison chart of composite services success rate with standard error.

The degree of user satisfaction is also the main optimization goal of this article. The degree of user satisfaction is closely related to many factors, such as the number of requests for composite services reached per unit time, the number of service instances contained in each composite service, and the processing capacity of the container cluster set in the experiment. In this experiment, five scheduling algorithms are used in the same experimental environment to simulate simulation experiments on three composite service test sets, and then the success rate of each composite service test set is recorded, as shown in Figure 3.

By observing the above graph from a horizontal perspective, our algorithm can achieve the highest success rate compared to other scheduling algorithms. Vertically, with the increase in the number of composite services and service instances, the success rate of each scheduling algorithm after the completion of the composite service allocation is continuously reduced, but the reduction is different. Our algorithm is compared with the other four algorithms. It can be maintained in a relatively stable state, which ensures that the success rate of composite services is about 80% under different composite service test sets. The composite service success rate of Prioritized 3-DQN on the three data sets is about 4.82% higher than that of DQN. From the perspective of diversified loads, the Prioritized 3-DQN is more capable of making reasonable service scheduling decisions than other scheduling algorithms, thereby it increases the success rate of composite services and improves user QoS.

### 5.5 Resource utilization comparison

**FIGURE 4:**
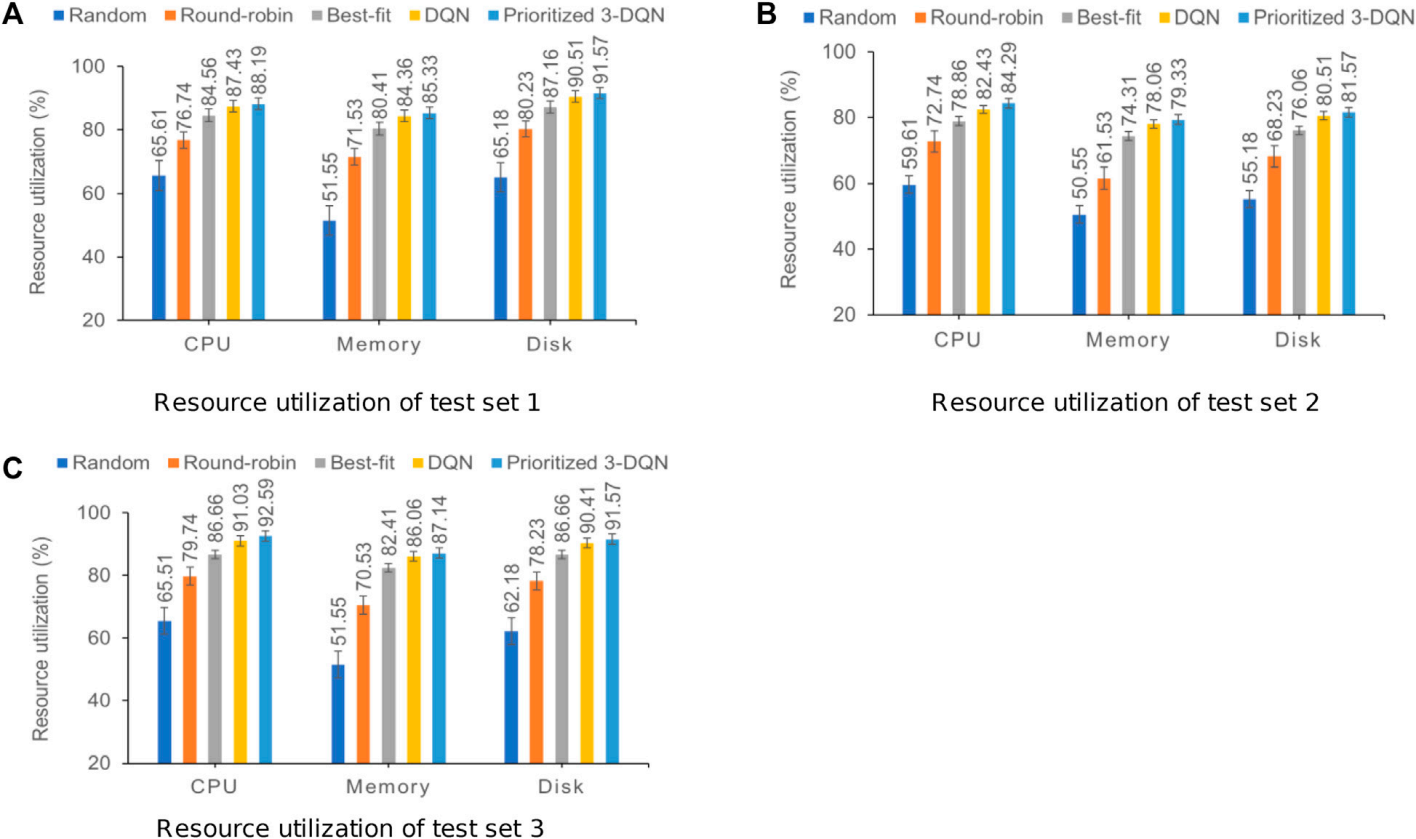
Resource utilization results of the three composite service test sets with standard error. **(A)** Resource utilization of test set 1. **(B)** Resource utilization of test set 2. **(C)** Resource utilization of test set 3.

In addition to completion time and user QoS, the resource utilization of a container cluster can also be used as one of the criteria for evaluating the performance of scheduling algorithms. This section compares and analyzes resource utilization from the three dimensions: CPU, memory, and disk. During the simulation experiment, the resource utilization rate of the container cluster was recorded after each service instance was scheduled, and the average result of each resource utilization rate was calculated after one round of scheduling was completed. Figure 4 shows the resource utilization results of the three composite service test sets.

The above three graphs show that our prioritized 3-DQN, DQN, and Best-fit algorithms are significantly higher than the other two algorithms in terms of resource utilization in the three dimensions, indicating that they can make full use of limited resources when scheduling service instances to complete the execution of composite services. When the Best-fit algorithm schedules service instances, it does not consider the data transmission relationship between services and the scheduling of subsequent service instances. It only schedules the current service instance to the container with the best performance and the shortest execution time. Therefore, the resource utilization in the three dimensions is lower than Prioritized 3-DQN and DQN. On the three data sets, the resource utilization of Prioritized 3-DQN on CPU, memory, and disk is about 1.39%, 1.11%, and 1.09% higher than that of DQN, respectively. The Prioritized 3-DQN is also higher than DQN in terms of resource utilization, indicating that Prioritized 3-DQN can make more reasonable scheduling decisions compared to DQN and has a more stable optimization capability under the same environment.

## 6 Conclusion

Cloud computing has brought great flexibility and cost-effectiveness to end-users and cloud application providers, and it has become a very attractive computing mode for various fields. With the continuous development of biological technology, massive biological data are continuously generated, and the requirements for data processing operation speed, computing power, and stability in practical applications also increase rapidly. Cloud computing has the characteristics of high-speed computing power, high storage capacity, and convenient use, which can meet the needs of biological research. At the same time, cloud providers provide security services to ensure the privacy and integrity of data. When biological samples are processed, each step needs to be supported and completed by cloud services. Between stages, biopharmaceutical companies realize data isolation by transferring data between services. Data quality plays a crucial role in the application effect of data, and the problem of data timeliness is one of the main factors affecting data quality. The timeliness of data can be improved synergistically by combining timeliness rules with statistical technical conditions or functional dependencies. How to use service scheduling strategy to improve service quality and resource utilization has become a key issue in cloud computing. This paper focuses on the core problem of service scheduling management in the container cloud platform. We proposed the composite service model under the modes of container instance (mixed reservation and on-demand), and we proposed the improved DQN algorithm as the scheduling algorithm of the composite service model in this paper. The simulation results show that, under the model presented in this paper, our 3-DQN algorithm is superior to the original DQN algorithm in terms of reliability and convergence. In addition, the algorithm can effectively reduce the completion time of the composite service and improve the user QoS and resource utilization in the container cloud environment.

The method proposed in this paper still has many defects for the actual cloud environment. From the results represented in the paper, the differences in completion time, composite service success rate, and resource utilization between DQN and Prioritized 3-DQN are small. The reason for the smaller difference may be that the scale of our experiments is relatively small. If the scale of the experiments is large, the advantages of Prioritized-3DQN may be more prominent. We also consider comparing Prioritized 3-DQN with the three algorithms used in this paper in the future. In addition, in the process of designing the composite service model in the container cloud environment, the energy consumption and resource cost of the cloud platform are not considered. We can do further research in future work.

## Data Availability

Publicly available datasets were analyzed in this study. This data can be found here: https://github.com/alibaba/clusterdata/blob/v2018/cluster-trace-v2018.
